# COVID‐19 and mental health: Impact on symptom burden in older people living with mental illness in residential aged care

**DOI:** 10.1111/ajag.13042

**Published:** 2022-02-07

**Authors:** Eleanor Curran, Liam Nalder, Digsu Koye, Jan Hocking, Brett Coulson, Sabah Khalid, Samantha M. Loi, Nicola T. Lautenschlager

**Affiliations:** ^1^ Aged Persons' Mental Health Program NorthWestern Mental Health Royal Melbourne Hospital Parkville Victoria Australia; ^2^ Academic Unit for Psychiatry of Old Age Department of Psychiatry Faculty of Medicine, Dentistry and Health Sciences University of Melbourne Parkville Victoria Australia; ^3^ Centre for Epidemiology and Biostatistics School of Population and Global Health The University of Melbourne Parkville Victoria Australia; ^4^ Methods and Implementation Support for Clinical Health Research Platform Faculty of Medicine, Dentistry and Health Sciences The University of Melbourne Parkville Victoria Australia; ^5^ Department of Neuropsychiatry Royal Melbourne Hospital Parkville Victoria Australia; ^6^ Melbourne Neuropsychiatry Centre Department of Psychiatry Faculty of Medicine, Dentistry and Health Sciences University of Melbourne Parkville Victoria Australia

**Keywords:** aged, COVID‐19, mental health, residential facilities

## Abstract

**Objectives:**

COVID‐19–related restrictions for residential aged care (RAC) have been significant. However, the mental health impacts for residents already living with mental illness remain poorly understood. In this study, we examined change in mental health symptom burden for this group and potential associations with clinical and contextual factors.

**Methods:**

We retrospectively reviewed medical records of patients of a specialist aged mental health clinical service for RAC. Change in symptoms (measured by the Neuropsychiatric Inventory, Nursing Home version [NPI‐NH]) between pre‐pandemic and two pandemic timepoints were analysed using Wilcoxon signed‐rank tests. Potential associations with baseline diagnosis or severity of ‘lockdown’ restrictions in RAC were assessed using linear regression.

**Results:**

Data from 91 patient files were included. The median NPI‐NH score slightly increased during wave one (baseline median NPI‐NH score = 17.0 [interquartile range, IQR: 10.0–27.0]; wave one median = 19.0, IQR: 8.0–30.0) and fell during wave two (Median: 15.5, IQR: 7.0–28.0), but changes were not statistically significant (all *p*‐values >0.05). Adjusting for age and gender, an association between neurocognitive disorder diagnosis and NPI‐NH score during wave one was statistically but not clinically significant (*p* = 0.046). No other significant associations were identified.

**Conclusions:**

Accounting for pre‐pandemic symptoms, we found no clinically relevant evidence of worsening mental health during COVID‐19 for a group of older people living with mental illness in RAC. This adds to evidence of relatively stable mental health in older people during the pandemic. Research and policy should consider underpinning mechanisms and emphasise patient‐ and carer‐centred interventions.

**Policy Impact**

This research provides the first Australian data regarding mental health impacts of the COVID‐19 pandemic for a specific, vulnerable population who have been subject to significant and sustained restrictions. This information can inform more tailored, effective and efficient policy for responding to the mental health impacts of the pandemic in residential aged care.

**Practice Impact**

This study will help aged care and mental health practitioners to direct resources and prioritise activities in this setting in the context of ongoing access and clinical capacity limitations.

## 1 INTRODUCTION

There has been ongoing concern regarding the mental health impacts of the coronavirus disease 2019 (COVID‐19) pandemic, including the restrictive measures required for infection control.^1^ However, real‐world data are essential in order to understand whether and how these anticipated consequences have borne out, particularly for vulnerable groups. This will be key to optimising policy and clinical practice to effectively and efficiently mitigate mental health impacts both during the ‘acute’ pandemic and in subsequent recovery phases.

Based on the relative severity of restrictions experienced, known access barriers to mental health interventions, historical epidemic data and evidence from other quarantine settings, people living in residential aged care (RAC) and older people living with existing mental health disorders (including neurocognitive disorders such as dementia) were identified as key vulnerable groups early in the pandemic.^2^, ^3^ However, subsequent studies in the general population have consistently found that increasing age may, instead, offer relative protection from adverse mental health impacts seen in younger age groups.^4^, ^5^ Findings have also been mixed regarding the experiences of adults living with existing mental health disorders, and of older people living in RAC. Some studies have reported increased neuropsychiatric symptoms (NPS) in dementia, loneliness and depression,^4^, ^6^, ^7^, ^8^ but others have found either no change in mental health symptom burden,^6^, ^9^, ^10^, ^11^ less change than in people without existing mental health disorders^12^ or improvement for some symptom types or NPS domains.^11^, ^13^, ^14^

In light of this indication that mental health impacts are more nuanced than initially anticipated, ensuring detailed understanding of specific patterns in key populations and subgroups is imperative. However, there has not yet been any investigation of pandemic‐related mental health effects for older people who both reside in RAC and live with existing mental health disorders. Prevalence of most mental health disorders is substantially higher in RAC than community‐dwelling older populations.^15^ This group also commonly lived with marginalisation or complex needs prior to the pandemic and has experienced some of the most severe and prolonged public health restrictions, or ‘lockdowns’.^16^, ^17^ Consequently, they may be particularly vulnerable to adverse pandemic‐related mental health effects, and policy and clinical‐service responses will require particularly careful planning and resourcing. Specific data to guide these responses are needed urgently.

To help address this knowledge gap, we analysed data from existing medical records in a specialist aged mental health clinical service, to obtain a longitudinal perspective of the impact of the COVID‐19 pandemic on mental health symptom burden for a sample of older people already living with mental health disorders in RAC. Given suggestions in the earlier literature that baseline diagnosis and the severity of isolation may influence impacts, we examined associations with these factors.^4^, ^12^ We hypothesised that symptom burden would increase, and that greater increase would be associated with longer and more severe restrictions.

## 2 METHODS

### 2.1 Design and setting

The study was conducted in a specialist aged mental health service (AMHS) providing outreach clinical care for individuals aged 65 years and older living in RAC in a defined geographical catchment area in Melbourne, Australia. Overall, Australia has experienced very few cases of COVID‐19 compared to most regions, particularly prior to the recent Omicron outbreak.^18^ However, following a small amount of community transmission in March‐April 2020, a more substantial ‘second wave’ occurred in Melbourne between July and September 2020, predominantly in RAC facilities in the catchment of this AMHS. Almost 75% of all COVID‐19–related deaths in Australia in 2020 occurred in RAC, the vast majority in Melbourne.^18^ Further, significant restrictions on visits and activities were mandated in all RAC facilities from at least 16^th^ March until early October 2020, and many RAC facilities imposed additional restrictions, particularly during the second wave. All relevant AMHS clinical work continued through this period, although most transitioned to telehealth. Hence, this AMHS has unique access to insights regarding the mental health effects of the pandemic in the Australian RAC context.

Prior to the pandemic, trained clinicians working with the AMHS collected mental health symptom data for all patients as part of routine clinical care. The same assessments were repeated during the first and second pandemic waves. From May 2020, clinicians were also asked to regularly assess the severity of restrictions at each patient's RAC facility. For this study, these data were extracted from medical records for all eligible AMHS consumers. Data extraction was independently undertaken by a psychiatrist and senior psychiatry trainee with the service (EC and LN), using a pre‐prepared tool. Discrepancies were resolved by discussion and consultation with a third author.

The project was approved by The Royal Melbourne Hospital human research and ethics committee (HREC reference: QA2020133). The study is reported in accordance with STROBE guidelines.^19^

### 2.2 Participants

Files of all AMHS patients between 1^st^ October 2019 and 10^th^ October 2020 were reviewed. Patient data was included where (1) primary diagnosis (according to Diagnostic and Statistical Manual of Mental Disorders, Fifth Edition [DSM‐5])^20^ was recorded by medical staff and (2) relevant psychiatric symptom burden rating had been assessed and recorded for at least two of the three timepoints of interest for the study (see below).

### 2.3 Variables

The primary outcome measure was the total score on the Neuropsychiatric Inventory, Nursing Home Version (NPI‐NH).^21^ This questionnaire assesses the frequency and severity of symptoms across 12 domains, such as delusions, hallucinations, agitation, anxiety and depression. The NPI‐NH has been validated in similar older populations (*α* = 0.67).^22^ It shows good correlation with other well‐established symptom rating scales. A total NPI score of >3 indicates clinically relevant symptoms, 4–12 indicates low symptom burden and ≥12 indicates high symptom burden.^23^ Maximum total score is 144.

We extracted NPI‐NH scores recorded from assessments at three timepoints of interest: (1) pre‐pandemic (baseline) – between 1^st^ October 2019 and 1^st^ February 2020; (2) immediately prior to relaxation of restrictions for the first COVID‐19 wave (wave 1) – between 30^th^ April and 15^th^ May 2020; and, (3) immediately prior to relaxation of second wave restrictions (wave 2) – between 27^th^ September and 18^th^ October 2020. Of note, while community restrictions were relaxed between the first and second pandemic waves, no or only minimal relaxation occurred in RAC.

We used a simple, bespoke measure of restriction severity (RS) in each RAC facility to reflect the severity of isolation experienced by patients, with face validity established through discussion with AMHS and RAC staff and the research team. Clinicians documented (weekly or as frequently as relevant and feasible) the following aspects of RAC facility restrictions for each patient: visitor restrictions, activity restrictions, movement restrictions. As variation in RS was largely confined to the second wave, an overall rating of RS for each RAC facility was given based on all entries between May and September 2020. RS ratings were defined as mild RS: no non‐essential visitors but alternative contact with family/carers/social network available (e.g. window visits or regular video calls) and at least 50% of usually scheduled internal activities continued; moderate RS: no or minimal alternative family/carers/social network visits and/or less than 50% of usually scheduled activities ongoing, but no restriction to individual rooms; severe RS: restriction to individual rooms for at least 3 weeks during wave two in addition to limited activities and social contact.

Demographic and clinical information extracted from files included age; gender; primary diagnosis (DSM‐5), categorised as neurocognitive disorder, schizophrenia spectrum disorder, affective/anxiety disorder, other disorder; comorbid medical and psychiatric diagnoses; any diagnosis of COVID‐19 during the study period; and reason for discharge from the service, in relevant cases.

### 2.4 Statistical analysis

Using conservative definitions based on the previous literature, we calculated that, for a significance level of alpha 0.05 and a power of 0.8, a sample size of 90 would detect a minimum clinically important difference of a six‐point change in the NPI‐NH total score (equivalent to a small to medium effect size).^24^, ^25^

Continuous variables were expressed as mean ± standard deviation (SD) or median ± interquartile range (IQR) as appropriate, and categorical variables were summarised as proportions. To assess the representativeness of the sample, demographic characteristics and diagnoses were compared with those of all patients seen by the service during the study period. Diagnostic category proportions were also compared between each assessment timepoint. Pairwise correlations between ratings at pre‐ and during‐pandemic timepoints were calculated for the sample as a whole to assess rank‐order stability of scores over time.

Shapiro‐Wilk test indicated NPI‐NH scores were skewed to the right; thus non‐parametric tests were used for the primary analysis. Due to the small sample size, analyses were only completed for total NPI‐NH scores and not for individual domains.

Change in the median total NPI‐NH score between each of the time points was assessed by Wilcoxon signed‐rank test. Friedman's test assessed overall differences across the total NPI‐NH scores.

Linear regression analyses then assessed associations between baseline diagnosis or RS and change from pre‐ to during‐pandemic NPI‐NH scores, following checks for assumptions. As RS is related to restrictions during wave 2, we examined the association between this variable and change between wave 1 and wave 2 NPI‐NH scores. We examined the effect of diagnostic category on the differences between pre‐pandemic baseline and both wave 1 and wave 2 NPI‐NH scores. As younger age and female gender have been consistently associated with worse pandemic‐related mental health, we adjusted for these variables.

Significance was set at *p* = 0.05 and all analyses were completed in STATA, version 16.1.^26^

## 3 RESULTS

In total, 91 patients had NPI‐NH scores recorded for at least two of the three timepoints of interest (78% of 116 eligible patients; i.e. individuals who were patients of BASICS during at least two of the three timepoints). Data for all three timepoints were available for 59 patients. Baseline scores were not available for seven patients, and wave 2 scores were not available for 25. Wave 1 scores were available for every patient. All missing data at wave 2 related to patient discharge (due to symptom improvement or death that was not related to mental health diagnosis). Unfortunately, no reasons for other missing data were documented in files. Twelve patients developed COVID‐19, and three of these patients died. COVID‐19 restriction severity levels were recorded for all patients.

Baseline demographic and clinical characteristics are summarised in Table 1. The mean age of the cohort was 75.9 (SD 7.9) years, 56% were female, and they resided in 63 different RAC facilities. Eight per cent had a primary diagnosis of a schizophrenia spectrum disorder, affective disorder or anxiety disorder, although comorbid neurocognitive disorder was present in over 50% of these patients. Baseline symptom burden was high (median NPI‐NH 17.0, IQR: 10.0–27.0). The sample had a significantly lower percentage of patients with primary diagnosis of a neurocognitive disorder (20%) relative to all patients seen by the service (28%) (χ^2^ (1) = 6.12, *p* = 0.013). The proportions of patients in other diagnostic categories were not significantly different to the wider AMHS (all *p*‐values >0.06). There were no significant differences between the proportion of samples at each timepoint on diagnostic category (all *p*‐values >0.3).

**TABLE 1:** Key demographic, diagnostic and contextual characteristics for the whole sample

Patient demographic characteristics (*n* = 91)	
Age, years, mean (SD)	75.9 (7.9)
Female, *n* (%)	51 (56.0)
COVID‐19 +ve, *n* (%)	11 (12.1)
Patient primary diagnosis category, *n* (%)	
Schizophrenia spectrum disorders	38 (42)
Affective/anxiety disorders	35 (38)
Neurocognitive disorder	18 (20)
Restriction severity experienced, *n* (%)	
Mild	38 (42)
Moderate	33 (36)
Severe	20 (22)

Note

COVID‐19 +ve: patients who had a confirmed diagnosis of COVID‐19 during the study period.

**FIGURE 1:**
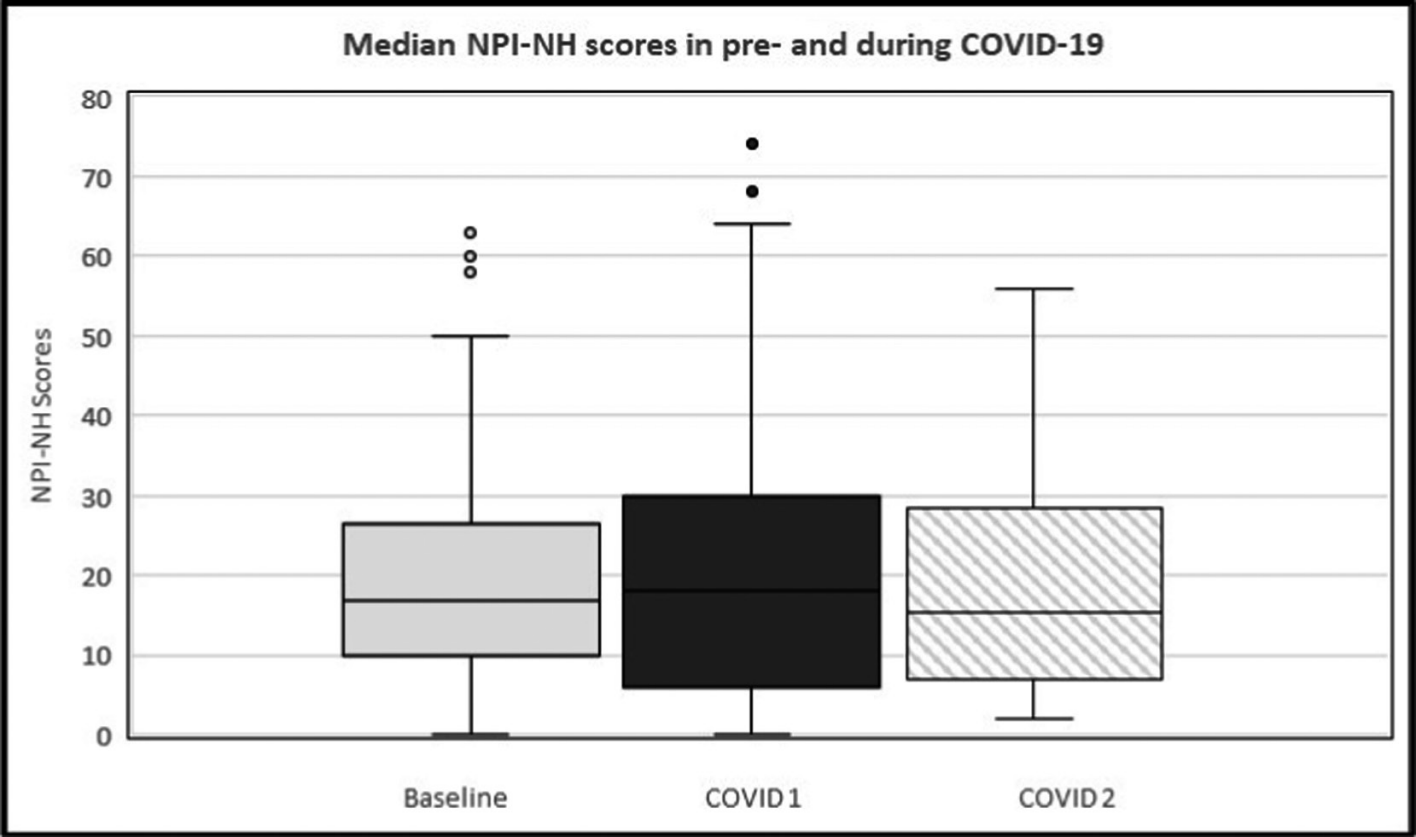
NPI‐NH scores in pre‐ and during‐pandemic timepoints. Median NPI‐NH scores and inter‐quartile range, at each timepoint of interest for the whole sample

Raw scores suggested a small increase in median total NPI‐NH scores during wave 1 (Med = 19.0, IQR: 8.0–30.0), followed by a small decrease to below baseline scores during wave 2 (Med = 15.5, IQR: 7.0–28.0) (Table 2 and Figure 1). However, these changes did not reach significance when comparing wave 1 to pre‐pandemic baseline scores (*z* = 1.06, *p* = 0.293); wave 2 to pre‐pandemic baseline scores (*z* = 0.10, *p* = 0.921); and wave 2 to wave 1 scores (*z* = −1.92, *p* = 0.054). Friedman test also indicated no significant difference overall across the total NPI‐NH scores (*p* = 0.081).

**TABLE 2:** Mental health symptom burden, as measured by the NPI‐NH, at each timepoint of interest for the whole group and subgroups according to diagnostic category and facility restriction severity level (wave 2 only)

Group/subgroup	Baseline NPI‐NH score		COVID‐1 NPI‐NH score		COVID‐2 NPI‐NH score	
	*n* (%)	Median (IQR)	*n* (%)	Median (IQR)	*n* (%)	Median (IQR)
Whole sample	84 (100)	17.0 (10.0–27.0)	91 (100)	19.0 (8.0–30.0)	66 (100)	15.5 (7.0–28.0)
Diagnostic sub‐groups						
Schizophrenia spectrum disorders	37 (44)	12.0 (10.0–24.0)	38 (42)	16.0 (7.0–27.0)	32 (48)	16.0 (7.0–28.0)
Affective/anxiety disorders	29 (35)	17.0 (10.0–23.0)	35 (38)	19.0 (6.0–28.0)	26 (40)	13.5 (8.0–22.0)
Neurocognitive disorders	18 (21)	26.0 (11.0–48.0)	18 (20)	31.0 (15.0–48.0)	8 (12)	25.5 (15.5–33.5)
RAC facility restriction severity (wave 2) sub‐groups						
Mild	N/A	N/A	38 (42)	16 (10.0–27.5)	25 (38)	17.0 (11.0–28.0)
Moderate	N/A	N/A	33 (36)	21.0 (7.5–31.5)	26 (39)	14.5 (5.25–26)
Severe	N/A	N/A	20 (22)	16.0 (6.0–30.0)	15 (23)	12.0 (7.0–27.5)

Note

Baseline: prior to COVID‐19 pandemic.

Abbreviations: COVID‐1, during the first wave of COVID‐19 in Melbourne; COVID‐2, during the second wave of COVID‐19 in Melbourne; IQR, interquartile range; N/A, not applicable; NPI‐NH, neuropsychiatric inventory, nursing home version; RAC, residential aged care.

Correlations between baseline, wave 1 and wave 2 NPI‐NH scores were all significant and of medium size, indicating good rank order stability over time and suggesting valid results.

Linear regression examined the effect of diagnostic category and of restriction severity level on changes in NPI‐NH scores between pre‐pandemic and during pandemic measures, adjusting for age and gender (Table 3). For the effect of diagnostic category (with schizophrenia spectrum disorders as reference), the estimated mean change in NPI‐NH scores from baseline to wave 1 for those with affective or anxiety disorders was −1.14 (95% CI: −9.18,6.89, *p* = 0.778), and the estimated mean change in NPI‐NH scores from baseline to wave 1 for those with neurocognitive disorders was 10.28 (95% CI: 0.18,20.38, *p* = 0.046). Diagnostic category was not significantly associated with mean difference in NPI‐NH scores between pre‐pandemic baseline and wave 2 (all *p*‐values >0.5). Finally, no significant association was seen between wave 2 restriction severity levels and mean difference in NPI‐NH scores between wave 1 and wave 2 (all *p*‐values >0.5) (Table 3).

**TABLE 3:** Linear regression models of NPI‐NH scores for anticipated predictors. Regression coefficients represent the effect of the predictor variable on change in NPI‐NH scores between specified timepoints

Factor	Beta (95% CI)	*p*‐value
Association between diagnostic category on difference between mean wave 1 and baseline scores		
Schizophrenia spectrum disorders)	Reference	
Affective/anxiety disorders	−1.14 (−9.17, 6.89)	0.778
Neurocognitive disorders	10.28 (0.18, 20.38)	0.046
Effect of diagnostic category on difference between mean wave 2 and baseline scores		
Schizophrenia spectrum disorders	Reference	
Affective/anxiety disorders	−1.66 (−9.40, 6.08)	0.669
Neurocognitive disorders	3.73 (−8.25, 15.71)	0.535
Association between restriction severity level on difference between mean wave 2 and wave 1 scores		
Mild	Reference	
Moderate	−2.29 (−9.30, 4.72)	0.516
Severe	−1.29 (−9.45, 6.86)	0.753

Abbreviation: NPI‐NH, neuropsychiatric inventory, nursing home version.

## 4 DISCUSSION

This study is, to our knowledge, the first examination of the effects of the COVID‐19 pandemic on mental health for older people living with mental health disorders in RAC and accounting for pre‐pandemic symptoms. Contrary to our hypotheses, we found no evidence of increased symptom burden during first two waves of COVID‐19, although the reduction in mental health symptom burden between pandemic waves approached significance. Further, an association between a primary diagnosis of neurocognitive disorder and greater increase in the NPI‐NH score during wave 1 was of marginal significance only, with wide confidence intervals indicating likely negligible clinical significance. We found no other significant associations between diagnosis or RS and overall change in symptom burden from pre‐pandemic through wave 2.

The absence of any meaningful differences or associations is itself an important finding. This is consistent with an emerging body of Australian and international evidence indicating that the anticipated mental health consequences have not eventuated for older people.^4^, ^5^, ^6^, ^10^, ^11^, ^27^ Recent studies including people living with existing mental health problems (older and general adult samples) similarly have not identified substantial deterioration, potentially suggesting that the pandemic has led to a greater prevalence of mental ill‐health but not to increased severity for existing disorders.^5^, ^6^, ^9^, ^12^ This study adds to the growing evidence by providing the first data indicating similar experiences specifically for older people living with a range of significant mental health disorders and in RAC.

Our findings also suggest that people living with neurocognitive disorders and NPS similarly did not experience significant symptom exacerbation. This is in contrast with some earlier findings^7^ but, importantly, is consistent with more recent and nuanced qualitative and quantitative data.^11^, ^14^

The reasons for these findings are unclear, and different factors may interact. Some authors have suggested greater resilience in older populations and related this to psychological mechanisms such as increased wisdom in ageing.^28^ However, as seen in both this and earlier reports, high baseline symptom burdens, limited movement and significant baseline isolation could suggest, instead, that the imposed restrictions caused little further deterioration as the impacts of these social/environmental determinants were already at saturation.^12^ The substantial pre‐pandemic symptom burden seen also suggests that earlier cross‐sectional studies may have over‐estimated pandemic‐related effects, highlighting the importance of longitudinal data.

In contrast, our analysis of only total NPI‐NH scores may have prevented identification of more nuanced findings such as concurrent exacerbation and improvement for different NPS domains. The absence of any association between RS and change in mental health symptom burden may also have been influenced by interventions and supports implemented during the study period. These may have meant the RS measure did not provide the expected reflection of social isolation. Even when experiencing severe restrictions, patients usually still had access to meaningful behavioural and social support. For example, our service increased the frequency of supportive contact (in‐person or using video‐call) with patients, staff and families, and extended the duration of episodes of care. The Australian Commonwealth Government also supported resident access to technology to maintain social and clinical connections. Such practical, person‐centred interventions to preserve social connectedness have previously been shown to mitigate adverse mental health impacts of more COVID‐19 restrictions and of isolation that is more broadly endemic in RAC.^12^, ^14^, ^28^, ^29^

### 4.1 Limitations

As with all observational studies, we cannot rule out that results reflected the impact of other factors, such as mitigation of adverse impacts by increased support from RAC staff or our AMHS. Further, the AMHS setting meant that the sample size was limited, inter‐rater reliability was not confirmed (although good rank order stability of scores over time argues against measurement bias), and results may not be generalisable to older people living in RAC who do not have sufficiently severe mental illness to warrant specialist involvement.

Most importantly, we did not examine mental health impacts in people referred after the pandemic was already underway. This meant that we could not assess the impact for people living with sub‐threshold or remitted symptoms prior to the pandemic. Some general population studies suggest that these groups may be more vulnerable to adverse impacts from lockdowns^13^ and their needs should be examined further in future work.

## 5 CONCLUSIONS

While small and necessarily pragmatic, this study provides an important additional insight into the mental health impacts of the COVID‐19 pandemic for vulnerable populations. It provides the first data specifically for people living with mental health disorders in RAC and engaged with specialist AMHS. It adds to related evidence by suggesting that mental health impacts of the pandemic for this group may be relatively limited and highlights the need for more research to explore more nuanced impacts and the underlying reasons for relative stability.

For clinicians and future planning, our results offer hope that mental health deterioration is not inevitable for these individuals during the pandemic, although mental health needs overall are substantial. Hence, optimising access to appropriate mental health care for these individuals, as well as resources and supports for RAC staff who primarily care for them and may be vulnerable to stress and burnout in the pandemic context, is crucial. Given ongoing pandemic challenges and increasing evidence both in pandemic and non‐pandemic contexts, it may be particularly fruitful for service planners to direct clinical resources and develop care models to support delivery of relatively simple person‐centred interventions with patients, families and, in particular, RAC staff. These should support social connectedness and aim to prevent or address distress in both patients and carers. This approach may offer benefits for a vulnerable and marginalised population that far outweigh and outlast the current pandemic.

## CONFLICTS OF INTEREST

No conflicts of interest declared.

## Data Availability

Data available on request due to privacy and ethical considerations.
